# Describing the indirect impact of COVID-19 on healthcare utilisation using syndromic surveillance systems

**DOI:** 10.1186/s12889-021-12117-5

**Published:** 2021-11-05

**Authors:** Claire F. Ferraro, Lucy Findlater, Roger Morbey, Helen E. Hughes, Sally Harcourt, Thomas C. Hughes, Alex J. Elliot, Isabel Oliver, Gillian E. Smith

**Affiliations:** 1 Field Service, UK Health Security Agency, Bristol, BS1 6EH UK; 2 National Institute of Health Research Health Protection Research Unit on Behavioural Science and Evaluation at the University of Bristol, Bristol, UK; 3 Real-time Syndromic Surveillance Team, Field Service, UK Health Security Agency, Birmingham, B3 2PW UK; 4 John Radcliffe Hospital, Oxford, Oxfordshire UK

**Keywords:** Syndromic surveillance, Healthcare utilisation, Viral, Epidemiology, Coronavirus, Pandemic

## Abstract

**Background:**

Since the end of January 2020, the coronavirus (COVID-19) pandemic has been responsible for a global health crisis. In England a number of non-pharmaceutical interventions have been introduced throughout the pandemic, including guidelines on healthcare attendance (for example, promoting remote consultations), increased handwashing and social distancing. These interventions are likely to have impacted the incidence of non–COVID-19 conditions as well as healthcare seeking behaviour. Syndromic Surveillance Systems offer the ability to monitor trends in healthcare usage over time.

**Methods:**

This study describes the indirect impact of COVID-19 on healthcare utilisation using a range of syndromic indicators including eye conditions, mumps, fractures, herpes zoster and cardiac conditions. Data from the syndromic surveillance systems monitored by Public Health England were used to describe the number of contacts with NHS 111, general practitioner (GP) In Hours (GPIH) and Out-of-Hours (GPOOH), Ambulance and Emergency Department (ED) services over comparable periods before and during the pandemic.

**Results:**

The peak pandemic period in 2020 (weeks 13–20), compared to the same period in 2019, displayed on average a 12% increase in NHS 111 calls, an 11% decrease in GPOOH consultations, and a 49% decrease in ED attendances. In the GP In Hours system, conjunctivitis consultations decreased by 64% and mumps consultations by 31%. There was a 49% reduction in attendance at EDs for fractures, and there was no longer any weekend increase in ED fracture attendances, with similar attendance patterns observed across each day of the week. There was a decrease in the number of ED attendances with diagnoses of myocardial ischaemia.

**Conclusion:**

The COVID-19 pandemic drastically impacted healthcare utilisation for non-COVID-19 conditions, due to a combination of a probable decrease in incidence of certain conditions and changes in healthcare seeking behaviour. Syndromic surveillance has a valuable role in describing and understanding these trends.

**Supplementary Information:**

The online version contains supplementary material available at 10.1186/s12889-021-12117-5.

## Background

The coronavirus pandemic (COVID-19) is currently causing an unprecedented global health crisis with significant impact on health and social care services and society worldwide. As of 21st June 2021, there have been over 178 million cases and 3.8 million deaths from SARS-CoV-2 globally [1]. The first cases in England were identified at the end of January 2020 [2, 3]. Widespread transmission in the UK led to a rapid increase in incidence during March reaching an initial peak of new cases in England in April [4]. During 2020, the daily number of deaths in England was at its highest at just over 1000 deaths in week 15 (6-12th April 2020).

**Fig. 1:**
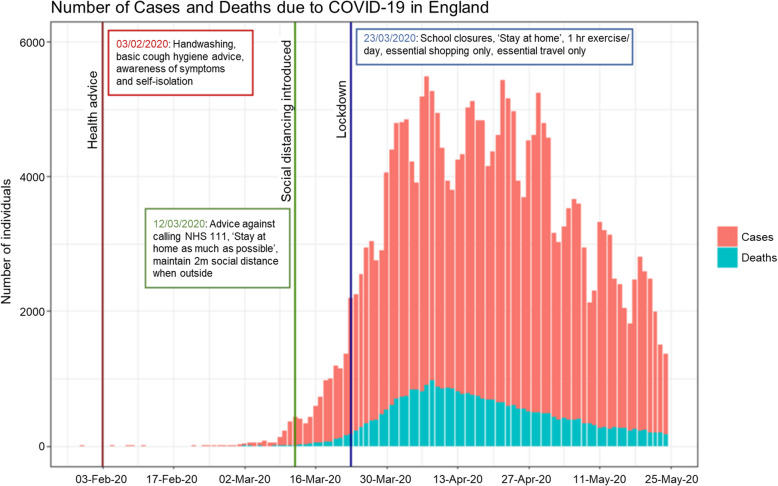
Number of cases and deaths from SARS-CoV-2 in England between January to May 2020. This graph shows the epidemiological curve of lab-confirmed cases and deaths (recorded within 28 days of positive test) due to SARS-CoV-2 in England (30/01/2020–24/05/2020), showing the introduction of non-pharmaceutical interventions at three significant dates, which are used as reference points in subsequent Figs. 2, 3, 4, 5, 6 [9]

The UK government’s COVID-19 public health information campaign was launched in early February encouraging individuals to increase handwashing and improve respiratory hygiene to ‘catch’ coughs and sneezes [5]. On the 12th March, the government announced a move from ‘contain’ to the ‘delay’ phase [6]. The announcement advised the public to use a new online version of the National Health Service (NHS) remote telephone health advice line; NHS 111 [7]. Primary care (general practitioner; GP) services in England started to conduct remote consultations in the first instance [8]. Stepwise restrictions on social mixing and daily life were introduced over the subsequent two weeks until the 23rd March when the first national ‘lockdown’ tightened restrictions further (Fig. 1). Ambulance services and emergency departments (EDs) continued to operate as usual throughout; however, significant changes in healthcare seeking behaviour were observed, potentially due to changes in need for urgent healthcare, possible fears of contracting the infection in healthcare settings, and a desire to ‘protect the NHS’ [10].

Public Health England (PHE; please note - on 1 October 2021 PHE was replaced by UK Health Security Agency and the Office of Health Improvement and Disparities) publishes weekly, publicly available, surveillance reports for the monitoring of the COVID-19 pandemic in England [11]. The COVID-19 surveillance activities reported are carried out across multiple data sources including laboratory confirmed cases; community outbreaks; internet surveys and search activity; hospitalisations; and mortality. These surveillance activities are further supplemented by syndromic surveillance of healthcare utilisation across five different areas of the NHS: NHS 111 (calls and online assessments); GP consultations, both In Hours (scheduled) and Out-of-Hours (unscheduled); ambulance calls; and ED attendances.

‘Syndromic’ surveillance is “the process of collecting, analysing and interpreting health-related data to provide an early warning of public health threats which require public health action” [12]. It is powerful at detecting changes in relatively non-specific symptoms or preliminary diagnosis information collected during routine healthcare provision. This can offer the near real-time ability to provide early warning, improve situational awareness, and monitor the emergence and spread of common infectious diseases and the public health impact of non-infectious diseases through the population. The important role of syndromic surveillance has been demonstrated during both major emergencies and mass gathering events, e.g. the 2009 global influenza pandemic [13, 14], during periods of extreme hot [15, 16] and cold [17] weather, and events such as the London 2012 Olympic and Paralympic Games [18]. COVID-19 has generated several new challenges for the syndromic surveillance systems, including: the impact of media reporting and social distancing measures on healthcare-seeking behaviour, changes in healthcare system delivery, and an increased demand for rapid analysis and dissemination of findings [19].

Understanding changes in healthcare demand is essential to interpret surveillance information. The PHE syndromic surveillance systems monitor a wide range of indicators of illness and healthcare utilisation. This paper aims to assess the indirect impact of COVID-19 and the introduction of non-pharmaceutical interventions (NPIs) on healthcare utilisation across different aspects of the NHS using syndromic surveillance. The syndromic indicators, chosen a priori*,* aim to explore the impact of mitigation measures such as increased handwashing, social distancing, and lockdown on healthcare utilisation across the different systems. In addition, herpes zoster and cardiac conditions were chosen as indicators which may indicate changes in decision making thresholds for individuals’ seeking healthcare during the pandemic.

## Methods

### Data sources

Data from the five PHE national syndromic surveillance systems were included in this study: remote health advice (NHS 111) calls and online assessments; GP In Hours consultations (GPIH); GP Out-of-Hours consultations (GPOOH); emergency department attendances (Emergency Department Syndromic Surveillance System (ED)); and ambulance calls [17]. The systems differ in their coding systems, range of indicators, and coverage across England (Table 1) [13, 21, 22]. The ED analysis was restricted to only those EDs which had reported throughout the study time period.

**Table 1:** Syndromic surveillance systems daily numbers during the peak pandemic period in 2020 compared to 2019

Syndromic surveillance system	Coverage	Description of indicator	Daily Mean N Weeks 13–22 in 2019	Daily Mean N Weeks 13–22 (Peak Pandemic) in 2020	Daily Mean N difference 2019 & 2020 (95% CI)^**1**^	Daily Mean Percentage Change 2019 & 2020 (95% CI)^**1**^
NHS 111	England-wide	**Total:** Number of calls made^2^	39,036	42,228	3192 (1889 to 4730)	+ 11.75% (+ 8.67% to + 15.06%)
		**Eye problems**: Patients calling and reporting symptoms of Eye or Eyelid Problems.	573	413	− 160 (− 115 to − 201)	−25.39% (− 20.07% to − 30.87%)
GP Out-of-Hours	System covers about 80% of England’s population	**Total**: Numbers of ‘contacts’ (either by telephone or in person)	26,741	23,257	− 3484 (− 2215 to − 4534)	−11.35 (−8.32% to − 14.07%)
		**Total with a Read code**: Number of contacts that have a ‘Read code’ (% of total contacts)	11,332 (42%)	8452 (36%)	− 2879 (− 2202 to − 3432)	− 24.02% (− 20.86% to − 26.87%)
		**Chest pain/myocardial infarction**: includes chest pain and acute myocardial infarction as a percentage of total contacts with a ‘Read code’	1.03%	1.60%	+ 0.57% (+ 0.51% to + 0.62%)	+ 60.93% (+ 52.82% to + 68.82%)
		**Eye irritation**: Eye irritation including conjunctivitis and eye infection as a percentage of total contacts with a ‘Read code’	0.75%	0.22%	−0.53% (− 0.50% to − 0.56%)	−71.06% (− 67.90% to − 74.27%
GP In Hours	System covers about 55% of England’s population	**Total**: Number of consultations at GP In Hours^3^	NA	NA	NA	NA
		**Mumps**: Clinical diagnosis of mumps reported per 100,000 population	0.100	0.055	−0.045 (− 0.027 to − 0.063)	−31.33% (− 11.45% to −54.49%)
		**Conjunctivitis**: Clinical diagnosis of conjunctivitis reported per 100,000 population	4.50	1.61	− 2.89 (− 2.70 to − 3.07)	−63.87% (− 62.75% to − 64.97%)
		**Herpes zoster**: Clinical diagnosis of Herpes zoster/shingles reported per 100,000 population	1.66	1.19	−0.46 (− 0.37 to − 0.55)	−27.00% (− 23.18% to − 30.92%)
Ambulance	System covers all 10 ambulance trusts in England	**Total**: Number of syndromic calls to 999 for ambulance service	14,349	13,783	− 566 (− 138 to − 1010)	−3.87% (− 0.89% to −6.92%)
		**Chest pain**: Calls where a person is described as experiencing chest pain or chest discomfort	1575	1220	− 356 (− 254 to − 456)	−22.37% (− 16.58% to − 28.74%)
Emergency Department (ED)	ED meeting criteria: submitting data on a daily consecutive basis, *N* = 100	**Total**: Number of attendances at ED	27916^4^	14819^5^	− 13,097 (− 12,516 to − 13,667)	−48.87% (− 45.03% to − 48.73%)
		**Total assigned a diagnosis**: Number of attendees at ED assigned a diagnosis (% of total)	22,348 (80%)	12,953 (87%)	− 9396 (− 8843 to − 9946)	− 41.95% (− 39.67% to − 44.25%)
		**Myocardial ischaemia**: Clinical diagnosis of myocardial ischaemia	379 (1.69%)	243 (1.87%)	− 136 (− 117 to − 155)	−34.88% (− 30.58% to − 39.26%)
		**Fractures**: Diagnosis of all fractures	1414 (6.33%)	723 (5.59)	− 690 (− 644 to − 738)	−48.67% (− 45.82% to − 51.63%)

The table describes the data sources, national coverage, and definitions of indicators from each of the five syndromic surveillance systems. Daily mean numbers per syndromic surveillance system and indicators in Weeks 13 to 22 of the peak pandemic period 2020 (Monday 23/03/2020 to Sunday 24/05/2020) are compared to the equivalent period in 2019 (Monday 25/03/2019 to Sunday 26/05/2019) presenting daily mean differences in number of contacts and as a percentage change with 95% confidence intervals

NA: Not applicable

^1^ 95%CI = 95% Confidence Interval. Calculated using boot-strapping method with R boot package, using bias corrected and accelerated (BCa) confidence intervals. 10,000 repetitions used

^2^ NHS111 calls exclude those callers with an immediate threat to life, abandoned calls, calls for information and calls which do not require triage

^3^ Total number of consultations at GP In Hours is not available. Consultations with a Read code are reported as a rate per 100,000 practice population for working days only (Monday – Friday, excluding bank holidays)

^4^ Total number of attendances between 01/01/2019 and 26/05/2019 inclusive was 2,129,878 across 100 Tier 1 EDs meeting criteria of submitting data on a daily consecutive basis; this is 32.02% of the total number of attendances from January 2019 to May 2019 inclusive published in national ED attendance records [20]

^5^ Total number of attendances between 01/01/2019 and 24/05/2020 inclusive was 2,105,082 across 100 Tier 1 EDs meeting criteria of submitting data on a daily consecutive basis; this is 40.55% of the total number of attendances from January 2020 to May 2020 inclusive published in national ED attendance records [20]

### Indicators

This study explored the trends in several conditions during the COVID-19 pandemic, considered likely to be affected by different components of the NPIs implemented in England (Table 1). These conditions were selected a priori, having been previously defined as ‘syndromic indicators’, specific to each surveillance system:

1. Eye condition indicators, monitored through GPOOH, GPIH, and NHS 111, were chosen because the incidence of infective conjunctivitis has been known to reduce due to handwashing campaigns during previous pandemics [23].
2. The introduction of social distancing measures would likely have an impact on other highly transmissible infectious diseases and therefore, knowing there had been a recent national outbreak of mumps across England, this was chosen as an indicator, monitored through GPIH [24].
3. Attendances at EDs for fractures, which are severe enough to warrant attendance and therefore may be representative of true incidence, were chosen as a further indicator, as national lockdown restrictions are likely to have changed daily activities (for example, there might be fewer fractures associated with injuries occurring while commuting to work or playing team sports). Lockdown measures resulting in less travel, working from home, children not attending school and closure of the night-time economy, may have resulted in a change to the distribution in time, gender, and age-groups of those attending ED for fractures. Therefore, ED attendances for fractures during the pandemic, broken down by gender, were analysed by hour of the day and day of the week.
4. To investigate changes in healthcare utilisation, the indicator for herpes zoster was chosen, monitored through GPIH, as incidence of a re-emergent viral infection was considered unlikely to be significantly affected by the COVID-19 pandemic NPIs, but rather indicate changes in individuals’ decision making threshold for seeking healthcare.
5. Cardiac indicators (chest pain/myocardial infarction indicator (GPOOH), myocardial ischaemia (ED), and chest pain (ambulance calls)) were chosen as indicators of severe life-threatening conditions which would usually require healthcare, therefore demonstrating changes in healthcare seeking behaviour during the pandemic.

### Time period

Trends of the selected indicators between Wednesday 1st January 2020 to Sunday 24th May 2020 inclusive were compared visually with Tuesday 1st January 2019 to Friday 24th May 2019. Key dates when public health messages or other interventions were introduced were considered (Fig. 1). The nine-week (63-day) period of the first national lockdown (week 13 – week 22: Monday 23rd March 2020 to Sunday 24th May 2020) was chosen to compare epidemiological trends at the peak of the COVID-19 pandemic with the equivalent period, matched by day of the week, in 2019 (week 13 – week 22: Monday 25th March 2019 to Sunday 26th May 2019).

### Analysis

Data on the number of total attendances and indicator-specific attendances are presented differently for each syndromic surveillance system: as a rate per 100,000 registered population for GPIH; as the percentage of total calls for NHS 111; as counts for ambulance calls (for syndromic type calls only, not all ambulance calls) and ED; and as a percentage of total contacts with a ‘Read code’ for GPOOH. Read codes are a coded thesaurus of clinical terms which record findings and procedures in NHS settings [25]. Seven day moving averages (adjusted for bank holidays) were calculated for all systems. The GPIH seven day moving average additionally adjusts for the number of working days in each week [26].

To investigate the overall number of attendances, consultations, or contacts for each surveillance system over the period of interest, trends were visually inspected to describe the total numbers and seven day moving averages over time in 2020 compared to 2019, for NHS 111, GPOOH, ambulance calls, and ED (these data were not available for GPIH). Daily mean number of attendances, consultations, or contacts during the nine-week peak period in 2020 and 2019 (weeks 13 to 22) and the daily mean differences were calculated and confidence intervals estimated by bootstrap sampling (10,000 repetitions used) using the bias corrected and accelerated (BCa) methodology to minimise bias. Boot-strapping enabled estimation of confidence intervals for differences, including rates and counts, without requiring modelling assumptions about underlying probability distributions.

Plots were produced describing the total daily number and seven day moving average of reported calls or consultations for indicator conditions monitored by each system, for 2020 compared to 2019, to explore how their epidemiology changed throughout the COVID-19 pandemic.

All analyses were conducted in R (version 3.5.2) using tidyverse, boot, and ggplot2 packages [27–30].

## Results

This study used PHE syndromic surveillance data from Wednesday 1st January 2020 to Sunday 24th May 2020, and Tuesday 1st January 2019 to Friday 24th May 2019 (one day fewer due to 2020 being a leap year). These figures represent the data routinely available through the syndromic surveillance systems, but for some systems this is not necessarily representative of the whole of England (Table 1). The total number of NHS 111 calls was 5,630,117 for this time period in 2019, and 6,175,249 in 2020, an increase of 9.68%. The total number of GP Out-of-Hours contacts was 3,803,553 in 2019, and 3,665,455 in 2020, a decrease of 3.63%. The number of GP Out-of-Hours contacts assigned a Read code was 1,634,938 in 2019 (43% of contacts), and 1,331,478 in 2020 (36% of contacts). The daily mean GPIH registered practice population was 23,998,209 in 2019, and 39,455,662 in 2020, an increase of 64.41%. The number of ambulance calls was 2,100,527 in 2019, and 2,105,082 in 2020, a slight increase of 0.22%. The total number of attendances at the 100 emergency departments included in the study was 3,909,796 in 2019, and 2,997,082 in 2020, a decrease of 23.34%. The number of ED attendances with a diagnosis was 3,138,470 in 2019 (80%), and 2,491,255 in 2020 (83%).

**Fig. 2:**
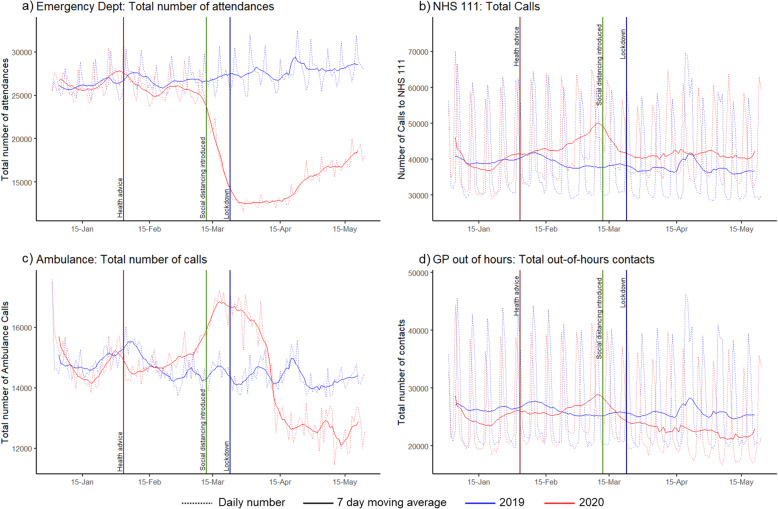
Overall total numbers of contacts in four different syndromic surveillance systems. These graphs show the daily (dotted line) and 7-day moving average (solid line) a) total number of attendances at emergency departments, b) total number of calls to NHS 111 as rate per 100,000 population, c) total number of calls to 999 for an ambulance, and d) total number of contacts with GP Out-of-Hours services

Daily trends in total activity across all five syndromic surveillance systems showed significant variation during the COVID-19 pandemic in England compared to equivalent months in 2019 (Fig. 2). The daily mean percentage changes in activity during the peak pandemic period in 2020 (Weeks 13 to 22) compared to the same period in 2019 are shown in Table 1. These showed the least difference for NHS 111 calls (12% increase; 95%CI: 9 to 15%) and GPOOH consultations (11% decrease; 95%CI: − 8 to − 14%). The total number of ambulance calls varied markedly throughout the pandemic period, increasing during the introduction of social distancing, before reducing during lockdown, such that the mean daily number during the peak pandemic period hides the marked variation seen in Fig. 2. The total number of attendances at ED nearly halved during the peak pandemic (daily mean percentage change: -49% (95%CI: − 45% to − 52%). (Trends in healthcare usage during the peak pandemic period in 2020 compared to 2019, stratified by weekdays, and weekend and bank holidays, for each syndromic surveillance system and indicators are available in Additional Table 1.)

**Fig. 3:**
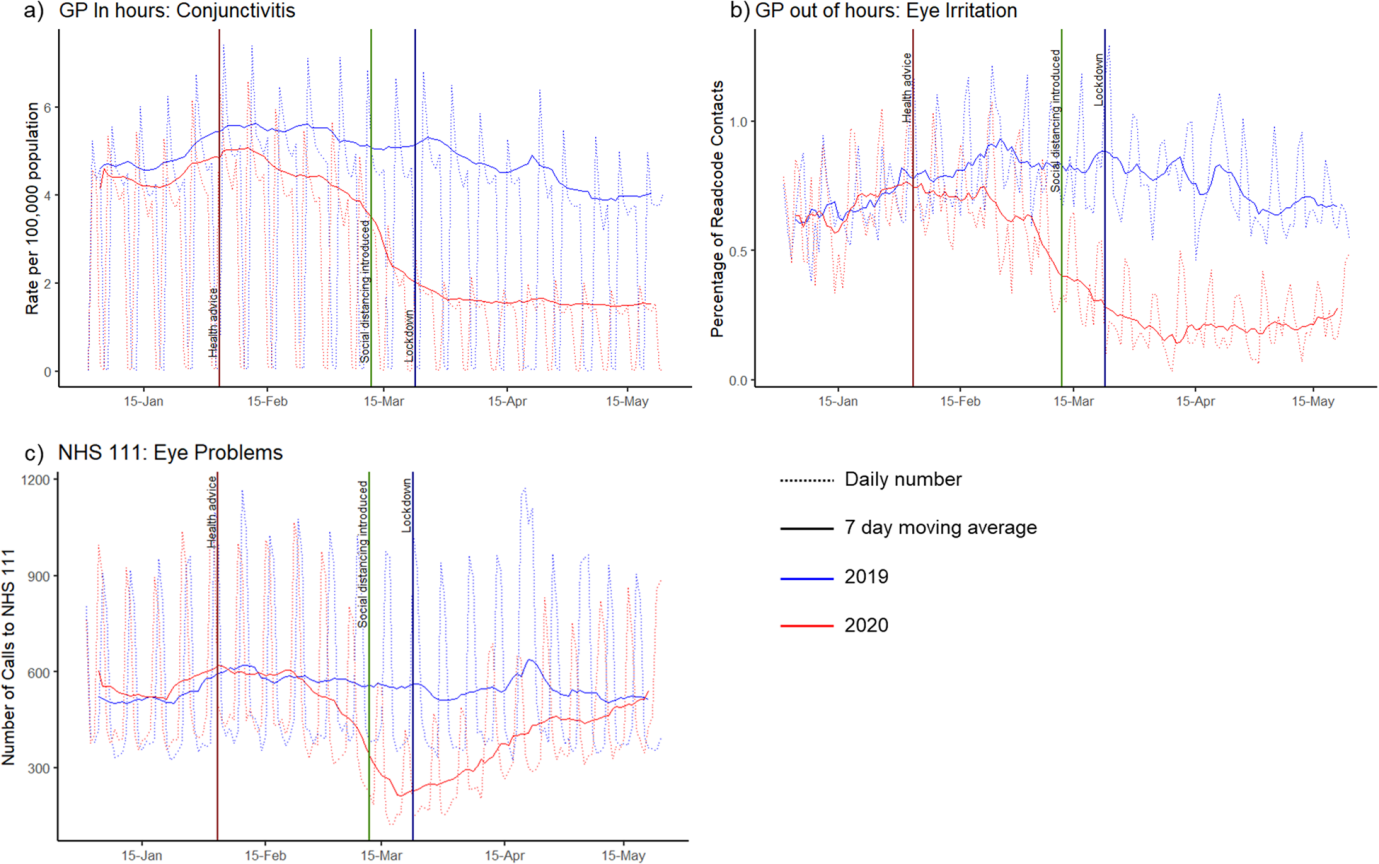
Numbers of contacts for eye conditions in three different syndromic surveillance systems. These graphs show the daily (dotted line) and 7-day moving average (solid line) a) number of GP In Hours consultations for ‘conjunctivitis’ per 100,000 population, b) number of GP Out-of-hours contacts for ‘eye irritation’ as a percentage of contacts with a Read code and c) number of calls per 100,000 population to NHS 111 for ‘eye problems’

Eye conditions, monitored through GPIH, GPOOH, and NHS 111, showed a sharp decline in mid-February (earlier than other syndromic indicators). GPIH conjunctivitis remained low throughout the 2020 peak pandemic period (64% reduction; 95%CI: − 63% to − 65%, compared to 2019 during peak pandemic period) as did GPOOH eye irritation (71% reduction; 95%CI: − 68% to − 74%). In contrast, though the number of calls to NHS 111 for eye problems initially reduced during March, call numbers returned to baseline (2019) by May, resulting in a smaller (25%; 95%CI: − 20% to − 31%) reduction across the entire peak pandemic period (Fig. 3).

**Fig. 4:**
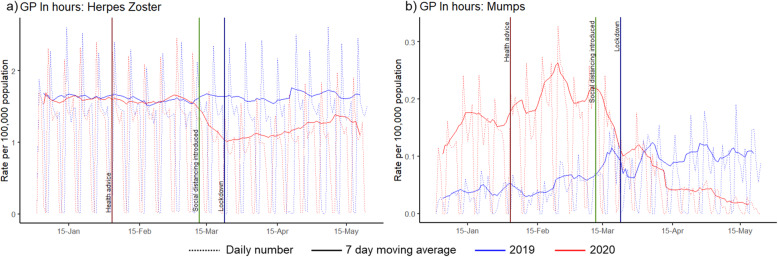
Numbers of contacts in GP In Hours syndromic system for two indicators. These graphs show the number of consultations per 100,000 population using GP In Hours daily syndromic surveillance data (dotted line) and 7-day moving average (solid line) for clinical presentation of a) Herpes Zoster and b) Mumps

GP In Hours mumps consultations showed a dramatic decline after the introduction of NPIs, following high activity early in 2020. During the peak pandemic period, the number of GP In Hours consultations for mumps showed a 31% (95%CI: − 11% to − 54%) reduction compared to 2019 (Fig. 4).

**Fig. 5:**
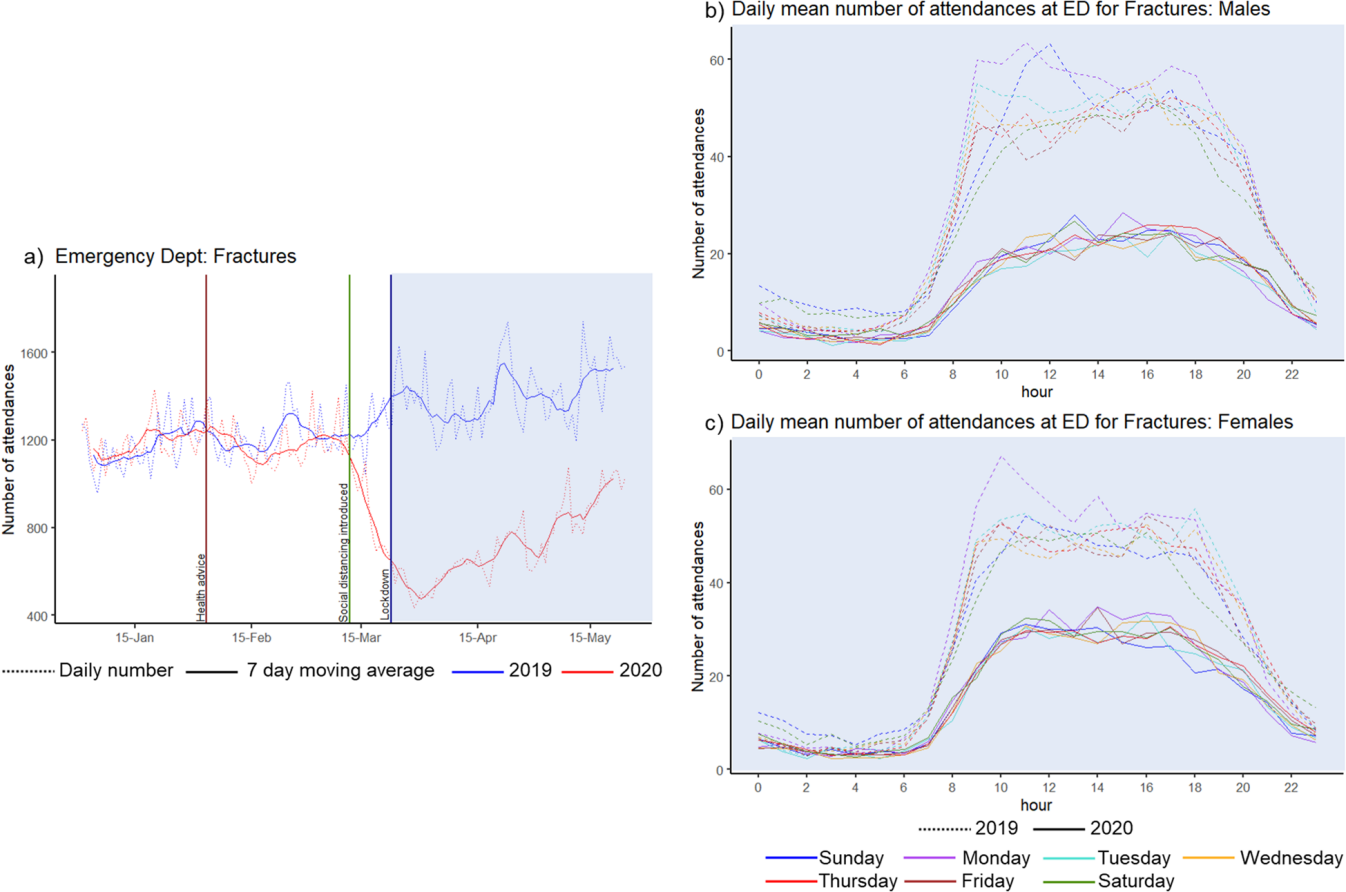
Number of attendances at emergency departments with a diagnosis of fracture by day and hour. These graphs show the a) number of daily (dotted line) and 7-day moving average (solid line) number of attendances at emergency departments and, for the period of ‘peak lockdown’ highlighted in blue (23/03/2020–24/05/2020 compared to 25/03/2019–26/05/2019), the b) daily mean number of attendances by hour of day, day of week (indicated by colour) in males in 2019 (dotted) compared to 2020 (solid) and c) daily mean number of attendances by hour of day, day of week (indicated by colour) in females in 2019 (dotted) compared to 2020 (solid)

Fractures presenting to EDs showed a sharp decline from the 12th March 2020 when COVID-19 restrictions were introduced, before starting to increase during May (Fig. 5a). During the peak pandemic period, there was a 49% (95%CI: − 46% to − 52%) reduction in attendance at ED for fractures compared to the same period in 2019. In 2019, weeks 13 to 22, the mean number of females attending ED with a fracture was highest on Monday mornings and for males there were peaks on both Sunday late morning and Monday morning. The trends of ED attendances by hour of day and day of week for the peak pandemic period in 2020 demonstrated that there was no longer any weekend effect, resulting in similar patterns of attendance across each day of the week for both males and females (Fig. 5b, c).

GP In Hours consultations for herpes zoster showed a decline following the interventions in March and an overall 27% reduction (95%CI: − 23% to − 31%) in consultations during the peak pandemic period compared to 2019 (Fig. 4).

**Fig. 6:**
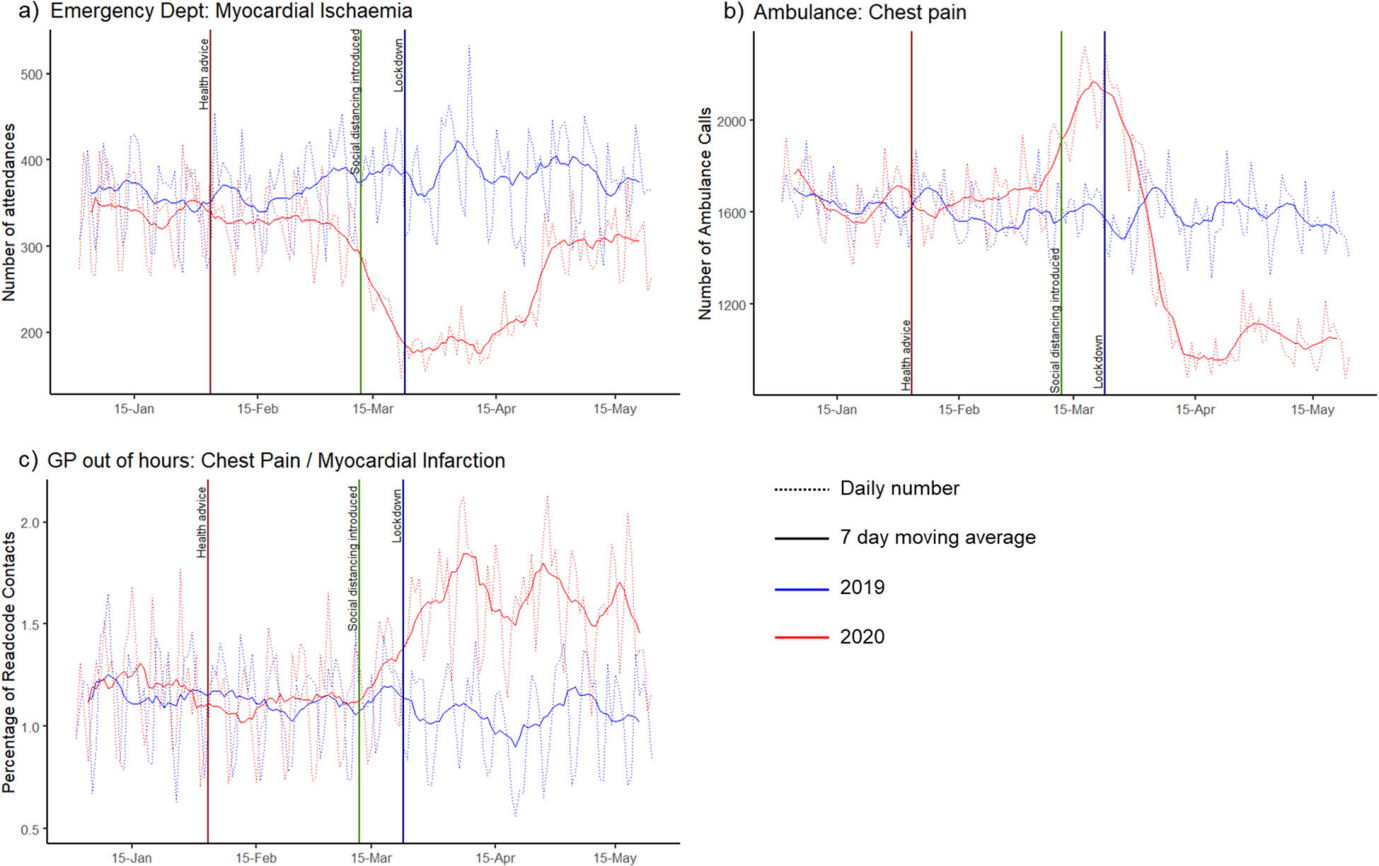
Numbers of contacts for chest-pain syndromes in three different syndromic surveillance systems. These graphs show daily syndromic surveillance data (dotted line) and 7-day moving average (solid line) for a) number of attendances with ‘Myocardial Ischaemia’ at emergency departments b) number of calls to 999 for an ambulance due to ‘Chest Pain’, and c) number of GP Out-of-Hours contacts for ‘Chest Pain / Myocardial Infarction’ as a percentage of contacts with a Read code

ED attendances for myocardial ischaemia also showed a marked decline following the interventions in March, with a 35% reduction (95%CI: − 31% to − 39%) in attendance for the peak pandemic period in 2020 compared to 2019 (Fig. 6). Opposing trends were observed for chest pain in ambulance calls and chest pain/myocardial infarction in GPOOH.

## Discussion

There were significant changes to healthcare utilisation in the first few months of the pandemic response in England. Total numbers of contacts with healthcare services in England, as demonstrated through the NHS 111, Ambulance, and both GP syndromic surveillance systems, increased in early March. A marked decline in activity, compared to 2019, was then observed across all syndromic systems (including ED) around the time of the introduction of COVID-19 NPIs and restrictions to activities in mid to late March. Similar rapid reductions in ED visits have also been reported in the USA, where ED visits declined by 42% during the early COVID-19 pandemic period, particularly among children and females, and in Thailand where ED visits overall declined by 36% [31, 32]. A systematic review summarising the impact of COVID-19 pandemic on healthcare utilisation from 81 studies across 20 countries published before 10 August 2020 found a median 37% reduction in services overall compared to pre-pandemic trends [33].

Healthcare contacts for different conditions displayed different trends during the peak pandemic period. Eye conditions showed a decline in activity earlier than other indicators, which may be explained by healthcare utilisation changing most significantly for milder conditions, or this may reflect a true decrease in incidence due to changes in hygiene behaviour reducing the spread of infectious conjunctivitis [23]. The persistence of low activity of eye conditions may be due to the closure of schools and nurseries during the peak pandemic period. Improvements in hygiene behaviour, combined with social distancing and in particular the closure of universities in mid-March, may also explain the dramatic reduction in mumps consultations in GPIH, bringing a rapid end to the national mumps outbreak of 2019/2020 that was concentrated particularly in university student populations [24].

The trends in attendances at ED for fractures, a high acuity condition that is more likely to represent true incidence, showed overall reduced numbers during the peak pandemic study period. Other data sources for incidence of trauma during COVID-19 show the same trend: referrals to specialist spinal surgery decreased significantly compared to the same period in 2019, most apparent for the number of high-energy traumatic presentations, which declined by 72% (*p* < 0.002) [34]. This is likely because of significant changes to daily activities, including the closing of schools and less participation in team sports, during the peak pandemic period. Data from the UK Government Department for Transport show mean car traffic use during the peak pandemic (weeks 13 to 22) was reduced by 61% compared to the baseline first week of February 2020 [35] likely resulting in fewer road traffic collisions. The trends in attendances for fractures show a loss of the weekend effect which may be partly due to a reduction in team sports and a reduction in alcohol-related attendances, as a consequence of the closure of the service industry, which has previously been shown to account for a peak of 72% of attendances in males in early hours at weekends [36]. The significant decline in ED attendance due to myocardial ischaemia during the COVID-19 pandemic was of concern to clinicians especially given the association between acute infection and myocardial infarction [37]. Further analysis of ED data showed the reduction in attendance was greatest at lower levels of acuity, and yet level 1 acuity ‘immediate’ attendances still reduced by 31% during COVID-19 [10]. This is in keeping with other reports on the reduction in the number of stroke patients attending, where the greatest decline was among mild severity stroke [38] and systematic review finding larger reductions in utilisation among people with milder spectrum of illness [33]. These dramatic declines in attendances at ED prompted public health messaging urging patients to continue to seek medical care as required [39].

A strength of this study is its use of the breadth of data across five national syndromic surveillance systems to demonstrate changes in healthcare utilisation across several healthcare systems and for a wide range of mild to severe conditions in England over time. This routine, real-time monitoring of syndromic indicators of disease provides a greater understanding of trends in healthcare utilisation, particularly for infectious diseases [13, 40]. The ability of ED data to add insight into changing trends in attendances by hour of day and day of week provides an added benefit of working with this syndromic data. This has previously been used to analyse the impact of sporting events on the timing of ED attendances [18, 41].

One limitation of the study is that some changes in indicator trends may result from modifications that have been made to the underlying health care systems to improve patient management rather than actual changes in activity. However, experience and detailed knowledge of the surveillance system and data by those undertaking the surveillance can help to minimise these limitations. For example, opposing trends in chest pain syndromes were observed throughout the first lockdown, however, the marked reduction in chest pain in the Ambulance system followed the introduction of a ‘pandemic’ chief complaint pathway (the basis of a newly defined COVID-19-like syndromic indicator) in early March. This suggested that the earlier peak in chest pain may have been detecting increased incidence of COVID-19 and/or anxiety, rather than cardiac-related chest pain. A COVID-19-like indicator was not introduced in the GPOOH system and therefore the chest pain/myocardial infarction indicator may have continued to capture some COVID-19 activity (albeit a small increase in percentage of Read code consultations). This demonstrates the importance of detailed interpretation from experts who understand the data’s strengths and limitations. Additionally, it is difficult to disentangle behavioural changes in healthcare usage and changes in disease incidence. This was in part minimised by exploring a range of different surveillance systems and indicators, for both mild and more severe health conditions.

This study improves our understanding of how healthcare usage changed during the first national lockdown, which began on the 23rd March 2020, both in total and for a range of syndromic indicators. This understanding is key when interpreting syndromic surveillance data and considering healthcare utilisation during the second and third national lockdowns (which were introduced in November 2020 and January 2021 respectively), when the NHS continued much of its routine healthcare provision and public health messaging about the importance of attending healthcare when needed was generally better emphasised [42]. Future work could describe how trends in syndromic indicators changed throughout summer 2020 when fewer restrictions were in place. Future sub-analyses could include exploring the trends in attendances for fractures by gender and age-groups and myocardial ischaemia by severity of illness markers. Importantly, our increased knowledge of how trends in syndromic surveillance are changing throughout the COVID-19 pandemic will be important when interpreting trends in other conditions, for example the monitoring of influenza throughout the seasonally expected peak influenza activity season, against a background of changing healthcare usage, behaviours, and incidence of other conditions.

## Conclusions

This descriptive analysis of syndromic surveillance system data demonstrates the significant impact of COVID-19 on England’s health, National Health Service and society. The restrictions introduced to contain and delay the spread of COVID-19 and to ease pressure on the health service resulted in a combination of a likely true decrease in incidence and a change in healthcare seeking behaviour for certain conditions. Syndromic surveillance systems have a valued and important role in describing and interpreting these trends.

## Supplementary Information

**Additional file 1: Table S1.** Daily mean percentage change between 2019 and 2020 peak pandemic period by indicators.

## Data Availability

The datasets analysed in this study are not publicly available. Syndromic surveillance data are collected and held by PHE for surveillance purposes, with no provision for use other than national surveillance outputs. Further information about accessing PHE data through the Office for Data Release can be found here: https://www.gov.uk/government/publications/accessing-public-health-england-data/about-the-phe-odr-and-accessing-data

## Abbreviations

NHS: National Health Service
PHE: Public Health England
GP: General Practitioner
GPIH: GP In Hours
GPOOH: GP Out-of-Hours
ED: Emergency Department
NPIs: Non-pharmaceutical interventions
BCa: bias corrected and accelerated
