# Supplementary material for: Describing the indirect impact of COVID-19 on healthcare utilisation using syndromic surveillance systems
Source: BMC Public Health. 2021 Nov 5;21:2019. doi: 10.1186/s12889-021-12117-5 (PMC8571013; doi:10.1186/s12889-021-12117-5)
Supplement: Supplementary file 1 — Additional file 1: Table S1. Daily mean percentage change between 2019 and 2020 peak pandemic period by indicators. [file 12889_2021_12117_MOESM1_ESM.docx]

**SUPPLEMENTARY INFORMATION**

**Describing the indirect impact of COVID-19 on healthcare utilisation using syndromic surveillance systems**

**Authors**

Claire F. Ferraro (corresponding author)

Lucy Findlater

Roger Morbey

Helen E. Hughes

Sally Harcourt

Thomas C. Hughes

Alex J. Elliot

Isabel Oliver

Gillian E. Smith

**Additional Table 1: Daily mean percentage change between 2019 and 2020 peak pandemic period by indicators.**

| **Syndromic surveillance system** | **Coverage** | **Description of indicator** | **Daily Mean Percentage Change in 2019 & 2020 (95% CI)^1^** | | |
| --- | --- | --- | --- | --- | --- |
|  |  | **Description of indicator** | **Total** | **Weekday** | **Weekend and bank holidays** |
| NHS 111 | England-wide | **Total:** Number of calls made^2^ | +11.75% (+8.67 to +15.06%) | +18.55% (+16.48 to +20.66%) | -1.85% (-6.86% to +3.55%) |
|  |  | **Eye problems**: Patients calling and reporting symptoms of Eye or Eyelid Problems. | -25.39% (-20.07 to -30.89%) | -22.76% (-15.88% to -29.80%) | -30.67% (-22.76% to -38.74%) |
| GP Out-of-Hours | System covers about 80% of England’s population | **Total**: Numbers of ‘contacts’ (either by telephone or in person) | -11.35% (-8.32 to -14.07%) | -11.28% (-7.34% to -14.86%) | -11.52% (-8.14% to -15.12%) |
|  |  | **Total with a Read code**: Number of contacts that have a 'Read code' (% of total contacts) | -24.02% (-20.86% to -26.87%) | -25.23% (-21.14% to 29.10%) | -21.00% (-18.34 to -23.94%) |
|  |  | **Chest pain/myocardial infarction**: includes chest pain and acute myocardial infarction as a percentage of total contacts with a ‘Read code’ | +60.93% (+52.82% to +68.82%) | +53.12% (+43.57% to +61.82%) | +80.44% (+69.42% to 91.18%) |
|  |  | **Eye irritation**: Eye irritation including conjunctivitis and eye infection as a percentage of total contacts with a ‘Read code’ | -71.06% (-67.90% to -74.27%) | -76.08% (-73.07% to 79.29%) | -58.51% (-54.61 to -62.52%) |
| GP In Hours | System covers about 55% of England’s population | **Total**: Number of consultations at GP In Hours^3^ | NA | NA | NA |
|  |  | **Mumps**: Clinical diagnosis of mumps reported per 100,000 population | NA | -31.33% (-11.45% to -54.49%) | NA |
|  |  | **Conjunctivitis**: Clinical diagnosis of conjunctivitis reported per 100,000 population | NA | -63.87% (-62.75% to -64.97%) | NA |
|  |  | **Herpes zoster**: Clinical diagnosis of Herpes zoster/shingles reported per 100,000 population | NA | -27.00% (-23.18% to -30.92%) | NA |
| Ambulance | System covers all 10 ambulance trusts in England | **Total**: Number of syndromic calls to 999 for ambulance service | -3.88% (-0.89% to -6.92%) | -2.16% (-5.94% to +1.53%) | -7.32% (-2.92% to -11.94%) |
|  |  | **Chest pain**: Calls where a person is described as experiencing chest pain or chest discomfort | -22.37% (-16.58% to -28.74%) | -20.22% (-12.66% to -28.62%) | -26.67% (-19.48% to -35.91%) |
| Emergency Department (ED) | ED meeting criteria: submitting data on a daily consecutive basis, N=100 | **Total**: Number of attendances at ED | -46.87% (-45.03% to -48.72%) | -47.64% (-45.47% to -49.82%) | -44.96% (-41.51% to -48.41%) |
|  |  | **Total assigned a diagnosis**: Number of attendees at ED assigned a diagnosis (% of total) | -41.95% (-39.67% to -44.25%) | -42.81% (-40.12% to -45.53%) | -39.82% (-35.44% to -44.23%) |
|  |  | **Myocardial ischaemia**: Clinical diagnosis of myocardial ischaemia | -34.88% (-30.58% to -37.39%) | -36.70% (-31.51% to -42.07%) | -30.30% (-23.44% to -37.39%) |
|  |  | **Fractures**: Diagnosis of all fractures | -48.67% (-45.81% to -51.63%) | -49.45% (-46.48 to -52.46%) | -46.74% (-40.25% to -53.68) |

The table describes the data sources, national coverage, and definitions of indicators from each of the five syndromic surveillance systems. Total daily mean percentage change per syndromic surveillance system and indicators in Weeks 13 to 22 of the peak pandemic period 2020 (Monday 23/03/2020 to Sunday 24/05/2020) are compared to the equivalent time period in 2019 (Monday 25/03/2019 to Sunday 26/05/2019) stratified by weekdays and weekend and bank holidays.

NA: Not applicable

^1^ 95%CI = 95% Confidence Interval. Calculated using boot-strapping method with R boot package, using bias corrected and accelerated (BCa) confidence intervals. 10,000 repetitions used.

^2^ NHS111 calls exclude those callers with an immediate threat to life, abandoned calls, calls for information and calls which do not require triage.

^3^ Total number of consultations at GP In Hours is not available. Consultations with a Read code are reported as a rate per 100,000 practice population for working days only (Monday – Friday, excluding bank holidays).
